# Comparison of different starting gonadotropin doses (50, 75 and 100 IU daily) for ovulation induction combined with intrauterine insemination

**DOI:** 10.1007/s00404-012-2414-3

**Published:** 2012-06-27

**Authors:** Robert Streda, Tonko Mardesic, Vladimir Sobotka, Dana Koryntova, Lucie Hybnerova, Martin Jindra

**Affiliations:** 1 Sanatorium Pronatal, Na Dlouhé Mezi 4/12, Prague, Czech Republic; 2 Pronatal Plus, Prague, Czech Republic; 3 Department of Obstetrics and Gynaecology, University of Pardubice, Pardubice, Czech Republic; 4 Faculty of Health Studies, University of Pardubice, Pardubice, Czech Republic

**Keywords:** Intrauterine insemination, Clomiphene citrate, Gonadotropins, Follitropin beta, Pregnancy rate, Multiple pregnancy

## Abstract

**Purpose:**

To prevent multiple pregnancies the goal of ovulation induction by gonadotropins is to achieve only mono-follicular development. The most important issue is therefore to determine the starting dose. The aim of this study is to compare three different starting doses of follitropin beta to assess the lowest effective dose.

**Methods:**

We evaluated 92 cycles with ovarian stimulation for patients with unexplained infertility, anovulatory disorder or mild male factor. We prospectively divided patients into 50, 75 and 100 IU groups based on patients’ response to clomiphene citrate treatment.

**Results:**

We performed 87 intrauterine inseminations (95 % of cycles with ovulation induction). Five cycles were cancelled. We achieved 15 pregnancies; total pregnancy rate was 18 %. Pregnancy rate was 22, 10 and 28 % in 50, 75 and 100 IU follitropin beta groups. The average number of follicles was 2.0 ± 0.8, 2.2 ± 1.1 and 2.5 ± 1.8 (ns), total dose of gonadotropins (IU) 483 ± 192, 600 ± 151 and 830 ± 268 (*p* < 0.001), respectively. We observed one case of twins in 75 and 100 IU treatment group, as well (25 % risk).

**Conclusions:**

This study suggests that based on the dose which was chosen according to clomiphene citrate response, all treatment regimes were effective for ovulation induction. 50 IU of follitropin beta daily is the appropriate starting dose to support ovulation for clomiphene citrate-sensitive women. The disadvantage may be an increased risk of cycle cancellation due to low ovarian response. Daily doses 75 or 100 IU of rFSH increase total consumption of gonadotropins.

## Introduction

For unexplained infertility, mild male factor and anovulatory women who fail to ovulate or conceive with clomiphene citrate, gonadotropin ovulation induction combined with intrauterine insemination is used as the second-line therapy [8]. According to the available clinical studies about 15–20 % of pregnancies are twins and 5 % triplets or more [8, 17, 25]. Multiple pregnancies represent a relevant problem with high incidence of perinatal mortality and severe neonatal morbidity [23]. To prevent multiple pregnancies the goal of ovulation induction by gonadotropins is to achieve only mono-follicular development and ovulation [3, 20, 22]. Low-dose follicle stimulating hormone (FSH) regimens have succeeded in reducing the rate of multiple pregnancies to 2–6 % and the rate of ovarian hyperstimulation syndrome (OHSS), as well [23].

*The aim of ovarian stimulation* is to reach, but not exceeding, the threshold level of FSH [14]. In case of inappropriate ovarian response it is necessary to adjust the dose of FSH. Hugues in Ref. [15] concluded that ‘the choice of the FSH starting dose and the modality of subsequent dose adjustments are critical in controlling the risk of overstimulation’.

We hypothesized that starting dose 75 or 100 IU of recombinant follicle stimulating hormone (rFSH) comparing with the starting dose 50 IU of rFSH increases expected pregnancy rate due to achievement of higher number of follicles and reduction of the risk of cycle cancellation due to low ovarian response. On the other hand we expected mild increase multiple pregnancy rate and the risk of cycle cancellation due to high response.

*The objective of the study* was to determine the effect of different follitropin beta dose (50, 75 and 100 IU daily) on follicular development, thickness of the endometrium at the time of human chorionic gonadotropin (hCG) administration, total consumption of gonadotropins per cycle, pregnancy rate, risk of multiple pregnancy and the risk of OHSS.

## Materials and methods

Between March 2005 and June 2006 women with a history of infertility signed informed consent and were recruited for this prospective study. Inclusion criteria were as follows: infertility at least 1 year, patients at least 18 years of age but not >38 years, BMI between 18 and 30 kg/m^2^, basal serum FSH and luteinizing hormone (LH) ≤10 IU/l, normal uterine cavity, at least one patent Fallopian tube assessed by hysterosalpingography or laparoscopy with chromosalpingography, history of clomiphene citrate therapy and sperm count after swim-up ≥5 million/ml.

Based on patients history of response for clomiphene citrate treatment we divided the patients into 50, 75 and 100 IU follitropin beta group (the patients with proved response to clomiphene citrate one tablet daily were allocated into 50 IU follitropin beta group, the women with no response to clomiphene citrate one tablet daily but proved response to two tablets daily were enrolled into 75 IU follitropin beta group and finally the patients with proved response to clomiphene citrate three tablet daily were put into 100 IU follitropin beta group). We started administration of 50, 75 or 100 IU follitropin beta subcutaneously on day 3, patients underwent a transvaginal ultrasonography on day 9 (Toshiba Famio with a 7.5 MHz probe) and if necessary we adjusted the gonadotropin dose (if we observed follicles ≤12 mm in diameter than we increased the FSH dose by 25 IU). When at least one follicle achieved 18 mm in diameter and endometrium was at least 8 mm thick we administrated hCG 5,000 IU subcutaneously and after 36–38 h we performed intrauterine insemination with luteal phase support by micronised progesterone 200 mg daily vaginally. To minimize the risk of a multiple pregnancy, hCG was withheld if more than four follicles 14 mm in diameter were seen. The couple was advised not to have unprotected intercourse.

Statistical analysis was performed by χ² test and ANOVA (GraphPad Software). A value of *p* < 0.05 was considered statistically significant.

## Results

We evaluated a total number of 92 ovarian stimulations for couples with unexplained infertility, anovulatory disorder or mild male factor. We performed 87 intrauterine inseminations (95 % of cycles with ovulation induction).

Baseline and hormonal characteristics of patients are summarized in Table 1. No significant differences according to study groups were documented. We noted some differences (but not significant) in the frequency of patients with ovulatory disorders and mild male factor. Patients with ovulatory disorders were more frequent in the 100 IU group and patients with mild male factor in the 50 and 75 IU groups. Cycle characteristics according to treatment group are described in Table 2. No significant differences were observed in the need to adjust the initial gonadotropin dose. Total consumption of rFSH was significantly higher in women receiving starting dose 100 and 75 IU daily compared with starting dose 50 IU daily (*p* < 0.001). There was a slight, but not a significant, difference in number of follicles and thickness of endometrium between groups. Duration of treatment does not differ in the study groups. Five cycles were cancelled (8 %). Reasons for cycle cancellation are presented in Table 3. Table 4 shows outcome of treatment. Clinical pregnancy rate per completed cycle was 22, 10 and 28 %, respectively. No significant differences according to study group were documented. Total clinical pregnancy rate was 17 % (15 pregnancies in 87 completed cycles). Spontaneous abortion occurs in one case. We observed one case of twins in 75 and 100 IU treatment group, as well (25 % risk of multiple pregnancy). None of the patients developed ovarian hyperstimulation syndrome.

**Table 1:** Basic and hormonal characteristics of patients

	50 IU (*n* = 35)	75 IU (*n* = 42)	100 IU (*n* = 14)	*p* value
Age (years)^a^	30 ± 3	30 ± 3	29 ± 4	ns
Primary infertility (%)^b^	25 (71)	27 (65)	8 (58)	ns
Duration of infertility (years)^a^	1.5 ± 0.7	1.4 ± 0.7	1.9 ± 1.1	ns
Cause of infertility^b^				ns
Unexplained (%)	1 (3)	1 (2)	0 (0)	
Ovulatory disorder (%)	13 (35)	12 (28)	8 (57)	
Mild male factor (%)	21 (60)	29 (69)	5 (36)	
Endometriosis (%)	0 (0)	0 (0)	1 (7)	
FSH (IU)^a^	6.8 ± 1.5	6.6 ± 1.5	6.8 ± 1.2	ns
LH (IU)^a^	5.6 ± 2.0	5.7 ± 2.4	7.1 ± 2.3	ns

^a^Values are expressed as mean ± SD, ANOVA

^b^The frequency distribution analysis χ² test

**Table 2:** Characteristics of the stimulation cycles

	50 IU (*n* = 35)	75 IU (*n* = 42)	100 IU (*n* = 14)	*p* value
Initial dose adjustment *n* (%)^a^	5 (14)	2 (5)	2 (14)	ns
Mono-follicular cycle rate* n* (%)^a^	10 (29)	13 (31)	4 (28)	ns
Number of follicles more than 16 mm^b^	2.0 ± 0.8	2.2 ± 1.1	2.5 ± 1.8	ns
Thickness of endometrium (mm)^b^	8.5 ± 0.9	8.7 ± 1.4	9.1 ± 1.8	ns
Total consumption of rFSH (IU)^b^	483 ± 192	600 ± 151	830 ± 268	*p* < 0.001
Duration of cycle (days)^b^	13.3 ± 1.7	13.2 ± 1.7	13.4 ± 1.8	ns
Minimal total dose (IU)	300	375	500	
Maximal total dose (IU)	1,100	1,050	1,350	

^a^The frequency distribution analysis χ² test

^b^Values are expressed as mean ± SD, ANOVA

**Table 3:** Cancellation of the cycles

	50 IU (*n* = 35)	75 IU (*n* = 42)	100 IU (*n* = 14)	*p* value
Cancellation of the cycles* n* (%)^a^	4 (11)	1 (2)	0	ns
Reasons for the cycle cancellation:				
Low ovarian response* n* (%)^a^	3 (8)	0	0	ns
More than 4 follicles 14 mm* n* (%)^a^	0	1 (2)	0	ns
The follicle at side of salpingectomy* n* (%)^a^	1 (3)	0	0	ns

^a^The frequency distribution analysis was performed by χ² test

**Table 4:** Outcome of the treatment

	50 IU (*n* = 31)	75 IU (*n* = 42)	100 IU (*n* = 14)	*p* value
Clinical pregnancy rate *n* (%)^a^	7 (22)	4 (10)	4 (28)	ns
Abortions *n* (%)^a^	1 (3)	0	0	ns
Ongoing pregnancy rate *n* (%)^a^	6 (19)	4 (10)	4 (28)	ns
Twins *n* (%)^a^	0	1 (25)	1 (25)	ns

^a^The frequency distribution analysis was performed by χ² test

## Discussion

Intrauterine insemination is recommended as the first-line treatment for couples with unexplained infertility or male subfertility [6, 9, 17, 27]. The expected pregnancy rate of intrauterine inseminations in natural cycles is about 6.3 % [24]. Ovulation induction increases the number of available oocytes and pregnancy rate [24, 26, 27]. Costellos’s metaanalysis of randomized controlled trials [7] and Arici’s study [1] revealed increasing pregnancy rate with ovulation induction by clomiphene citrate than natural cycle combined with intrauterine insemination. Clomiphene citrate is recommended as the initial treatment for women with unexplained infertility, mild male subfertility and WHO group II anovulation [9, 20]. This treatment achieves ovulation in approximately 70 % of patients and pregnancy within 6 months in 35 % [11, 18]. Multifollicular growth explains high rate of multiple pregnancies after clomiphene citrate therapy—20 % risk of twins and about 5 % risk of triplets [8].

Costellos’s metaanalysis of randomized controlled trials [7] and Balash’s study [2] confirm increasing pregnancy rate with ovulation induction by gonadotropins than clomiphene citrate combined with intrauterine insemination. Randomized controlled trial [5] demonstrated that ovulation occurred in 70 % in the recombinant FSH group versus 66 % in the urinary FSH group, total dose of gonadotropins was 750 IU versus 1,035 IU and duration of treatment (10 days vs. 13 days), respectively. Other studies [10, 12, 13] compared recombinant gonadotropins versus urinary ones for ovulation induction combined with intrauterine insemination. In randomized study [10] Demirol proved pregnancy rate 25.9 % versus 13.8 %, respectively. The advantage of recombinant FSH is availability of small dose—50 IU daily—for self subcutaneous administration and safe profile of treatment. Based on studies [16, 22], which compared different gonadotropin preparations in intrauterine insemination, recombinant FSHs result in a better outcome for unexplained infertility than urinary gonadotropins. The daily doses of recombinant FSH from 50 to 100 IU were used [16].

For *unovulatory woman with PCO syndrome* treated with a starting dose of 50 IU rFSH there is necessary to increase a dose after 7–14 days of drug administration unless follicles are less than 12 mm in diameter [19]. A study conducted by Leader [18] compared dose increment 25 IU versus 50 IU. Similar with our results the 25-IU group had a higher incidence of mono-follicular growth (41.3 % vs. 21.8 %), ovulation (81.3 % vs. 60.3 %), lower total cumulative gonadotropins dose (887 IU vs. 984 IU). A dose increment 50 IU increases risk of overresponse from 5 to 20 %. About 5 % cycles are cancelled due to low ovarian response. Several randomized studies are available for unovulatory women with PCO syndrome, seeking the dose of gonadotropins. Both randomized studies [15, 18] conclude that a starting dose of 50 IU recombinant FSH is adequate. Current study with low dose step up protocol [3] demonstrated 90 % ovulation rate, 15 % pregnancy rate, one case of OHSS (1.3 %) and risk of multiple pregnancy 25 %.

For women treated for *unexplained infertility* or *mild male factor* who fail to ovulate or conceive with clomiphene citrate, non-randomized Calaf’s study [4] suggested starting dose 50 IU recombinant FSH daily. One retrospective study [21] compared 50 and 75 IU of recombinant FSH combined with intrauterine insemination for unexplained infertility and achieved pregnancy rate 10 %. The study concluded that minimal FSH stimulation (up to two follicles) combined with intrauterine insemination cycle may reduce the rates of twins and high-order multiple pregnancies without affecting overall pregnancy rates.

We can explain the fact that study [9] observed pregnancy rate 34.3 % in the gonadotropin group compared clomiphene citrate group for unexplained and male subfertility. This pregnancy rate was higher than in our study. Women in our study were treated with clomiphene citrate for 3 months before study occurred with additional pregnancy rate 12 % [24]. Our pregnancy rate was higher (17 % vs. 12 %) and risk of cycle cancellation due to low ovarian response lower (8 % vs. 13.2 %) compared with another study [8].

The limitation of our study should be number of cases in groups. Despite this fact we proved significant differences in total consumption of gonadotropins between groups. Moreover, we intentionally did not compare other available prognostic markers (for e.g. FSH level, anti-Mullerian hormone (AMH), inhibin B etc.) with results of our study. Another trial (probably multicenter) is necessary for comparing all parameters in the future.

In summary, our work aims to assess the lowest effective gonadotropin dose and to find simple and easy to measure prognostic value for later response to ovarian stimulation combined with intrauterine insemination. We hope that results of our study may be helpful in tailoring treatment to individual patients.

## Conclusions

This study suggests that based on the dose which was chosen according to clomiphene citrate response, all treatment regimes were effective for ovulation induction combined with intrauterine insemination. 50 IU of follitropin beta daily is the appropriate starting dose to support ovulation for clomiphene citrate sensitive women. The disadvantage may be an increased risk of cycle cancellation due to low ovarian response. Daily doses 75 or 100 IU of rFSH increase total consumption of gonadotropins. The choice of correct gonadotropin starting dose and its adjustment are important steps to minimize the risk of multi-follicular development, multiple pregnancy and OHSS.
